# Obstetrics and Gynecology Trainees Face Higher Musculoskeletal Demands than General Surgery Trainees in Simulated Laparoscopic Tasks—An Observational Study

**DOI:** 10.3390/healthcare13243223

**Published:** 2025-12-10

**Authors:** Zaibun Khan, Abdulwarith Shugaba, Matthew Davitt, Donna Shrestha, Joel E. Lambert, T. Justin Clark, Theodoros M. Bampouras, Lawrence D. Hayes, Helen E. Nuttall, Daren A. Subar, Nilihan E. M. Sanal-Hayes, Christopher J. Gaffney

**Affiliations:** 1 St Mary’s Hospital, Manchester University NHS Foundation Trust, Manchester M13 9WL, UK; 2 Lancaster Medical School, Health Innovation One, Lancaster University, Lancaster LA1 4AT, UKm.davitt@lancaster.ac.uk (M.D.); d.shrestha@lancaster.ac.uk (D.S.);; 3 BRIDGES Research Group, East Lancashire Hospitals NHS Trust, Blackburn BB2 3HH, UK; 4 Birmingham Women’s Hospital, Birmingham Women’s & Children’s NHS Foundation Trust, Birmingham B15 2TG, UK; 5 Department of Metabolism and Systems Science, University of Birmingham, Birmingham B15 2TT, UK; 6 School of Sport and Exercise Sciences, Liverpool John Moores University, Liverpool L3 3AF, UK; t.bampouras@ljmu.ac.uk; 7 School of Health and Society, University of Salford, Salford M6 6PU, UK

**Keywords:** laparoscopy, obstetrics, gynaecology, surgical training, electromyography, ergonomics

## Abstract

**Background/Objectives**: Laparoscopic surgery has become the pre-eminent surgical approach for performing general surgical and gynecological operations, but it can lead to musculoskeletal disorder in surgeons. This study aimed to investigate the musculoskeletal demands of completing four core laparoscopic skills tasks amongst Obstetrics and Gynecology (O&G) and General Surgery (GS) trainees, recognizing that differences between specialties may create different ergonomic and muscular demands. **Methods**: Ten O&G and ten GS trainees both performed the same four tasks to evaluate their core laparoscopic skills whilst using electromyography (EMG) to assess the physical demand of each task in the trainee groups as a percent of maximum voluntary contraction. **Results**: O&G trainees had significantly higher muscle activity when completing a hand–eye coordination (HEC) task (167.9 ± 63.8 vs. 92.5 ± 31.3%, *p* = 0.019), bimanual coordination (BMC) task (205.6 ± 80.7 vs. 106.9 ± 47.0%, *p* = 0.017), and suturing (267.7 ± 121.6 vs. 122.2 ± 33.0%, *p* = 0.016) task in the right trapezius and deltoid muscle groups compared to GS trainees. No difference was observed between trainee groups in the laparoscopic camera navigation (LCN) task (*p* = 0.438). **Conclusions**: There appears to be increased muscular activity in O&G compared to GS trainees during the same simulated laparoscopic tasks. The findings should inform training policy around the optimization of ergonomics to minimize the risk of musculoskeletal disorder.

## 1. Introduction

Most surgical procedures in Gynecology and General Surgery are performed using minimally invasive techniques such as laparoscopic surgery. Laparoscopic surgery is preferred because of its less invasive nature and enhanced patient recovery compared to traditional open surgery. However, there are unique challenges associated with laparoscopic techniques. Laparoscopic surgery requires a complex and demanding interplay of cognitive and motor skills [1].

Firstly, laparoscopic procedures often require surgeons to maintain static positions with sub-optimal posture and carry out repetitive movements, potentially leading to musculoskeletal disorder [2]. Our previous work has shown that the ergonomic and musculoskeletal demands of laparoscopic surgery are greater than robotic surgery [3], but there is scant reporting on whether surgical specialties using laparoscopic abdominal surgery use different ergonomics when completing similar tasks. Optimizing ergonomics during training is of key importance to mitigate musculoskeletal disorder and prolong career longevity [4]. While the ergonomic and musculoskeletal demands of laparoscopic surgery are well documented [5,6,7], there is limited evidence comparing these demands across surgical specialties while performing similar tasks. Understanding whether Obstetrics and Gynecology (O&G) and General Surgery (GS) trainees experience different physical and cognitive loads is critical for tailoring ergonomic training and reducing occupational injury risk.

In addition to ergometric challenges, laparoscopic surgery presents significant cognitive challenges [1]. These include the need for advanced hand–eye coordination, fast reaction times, sustained attention, and effective memory. During surgery, these cognitive demands are often intensified due to inherent challenges. These include restriction of the surgical instrument’s range of motion, as well as the use of visual systems which can limit depth perception [8]. These factors often make these procedures more challenging, especially for those who are early in their training journey. 

The cognitive load associated with conducting surgical tasks tends to vary depending on the surgeon’s level of expertise. More experienced surgeons tend to experience a lower level of mental and physical strain when performing these tasks, as evidenced by their lower NASA TLX scores. For example, one study demonstrated that consultant surgeons had significantly lower NASA TLX scores than trainees performing video-assisted thoracic surgery tasks [9]. Similarly, another study also showed that consultant surgeons had significantly lower NASA TLX scores than trainees performing laparoscopic cholecystectomy [10]. These findings suggest that more experienced surgeons are able to perform surgical tasks with less cognitive effort [11], which may contribute to improved performance and patient outcomes. This observational study primarily aimed to investigate the ergonomics of the same core laparoscopic tasks in O&G and GS trainees by measuring muscular electrical activity using surface electromyography (EMG). Additionally, cognitive demands were evaluated as our secondary outcome variable using electroencephalography (EEG). This aimed to help us understand how mental workload associates with surgical performance, such as hand–eye coordination, reaction times, attention, and memory. Specifically, cognitive load was indexed using alpha oscillations in the 8–12 Hz frequency range of the EEG signal. Alpha activity was selected as it is thought to support task-relevant cognitive functions, such as maintaining attention to a task and inhibiting any distracting information happening around us. By incorporating both physical and cognitive aspects, this study provides a comprehensive overview of the challenges faced by surgical trainees, helping to enhance surgical education and training.

## 2. Materials and Methods

A prospective cross-sectional study was conducted and reported according to the checklist for reporting of survey studies (CROSS). This work was completed as part of a larger project investigating differences in laparoscopic skills acquisition between O&G and GS trainees [10]. Ethical approval for this study was granted by Lancaster University Faculty of Health and Medicine Research Ethics Committee (FHMREC20033), and the study was prospectively registered at ClinicalTrials.gov (NCT05116332). Written informed consent was obtained before experimentation, and this study was conducted in accordance with the Declaration of Helsinki.

A subset of trainees (Table 1) from the study conducted by Khan et al. [12] completed four simulated laparoscopic tasks described in detail previously, whilst muscular activity was measured using EMG. In brief, the four core laparoscopic skills tasks were performed in a fixed ascending order of task difficulty: (task 1) laparoscopic camera navigation (LCN); (task 2) a hand–eye coordination task (HEC); (task 3) a bimanual coordination task (BMC); and (task 4) a laparoscopic suturing and intracorporeal knot placement task (KNOT). Task 1 mainly involved the use of the non-dominant hand, task 2 involved holding the camera with the non-dominant hand whilst using the dominant hand to perform the task, and tasks 3 and 4 both involved the simultaneous use of both hands for the completion of tasks. 

During the execution of the four simulated tasks, electromyography (EMG; primary outcome variable) activity was recorded bilaterally from the biceps brachii, lateral deltoid, trapezius, and latissimus dorsi muscles using surface EMG electrodes (Trigno, Delsys, Inc., Boston, MA, USA). These muscles were selected as they are those frequently reported as experiencing discomfort [13,14]. For the EMG acquisition, skin preparation, electrode placement location identification, and electrode placement were in accordance with SENIAM guidelines [15]. Prior to testing, each trainee performed a maximal isometric voluntary contraction (MVC) for 5 s and EMG was recorded (MVC EMG) to enable relative reporting of EMG (i.e., % MVC EMG). SENIAM guidelines for the MVC procedure were also applied for each muscle group [15], which involved ensuring that maximal contraction took place isometrically against resistance and with the muscle at an optimal length for force production. For the biceps brachii, this took place with the elbow flexed at a 90° angle, the forearm supinated, and resisting an elbow flexion load. The lateral deltoid procedure involved the shoulder being abducted at 90° whilst resisting a downwards force. The upper trapezius procedure involved performing a shrugging movement against downwards resistance, and the latissimus dorsi procedure involved the shoulder being at a 30° angle and extending against resistance. For all contractions, EMG was recorded at 2000 Hz for 120 s. To ensure consistent recording across all participants, for the LASTT tasks, recordings were collected during the trainees’ second attempt. For the KNOT task, EMG data collection commenced at the beginning of the task. The signal was filtered with a high-pass and low-pass filter set at 10 and 500 Hz, respectively, and subsequently smoothed using the root mean squared (RMS) values over 150 milliseconds using manufacturer’s software (EMGWorks, Delsys Inc., Boston, MA, USA). The upper band filter of 500 Hz was selected as opposed to 400 Hz as SENIAM recommendations [15] and modern guidelines for EMG systems [16] state that they are designed and built to reliably collect physiological data produced by motor units up to 500 Hz. This means that excluding signals with a bandwidth between 400 and 500 Hz may truncate physiologically meaningful data. This is also recommended by other research that suggests that using a filter of 500 Hz allows for the EMG’s signal power to be maintained [17].

Similarly to the EMG monitoring, EEG data (secondary outcome variable) was captured for 120 s during the performance of the tasks. For the tasks, the EEG recording occurred during the trainees’ second attempt, thereby maintaining uniformity among all participants. For the KNOT task, we started collecting EEG data from the beginning of the task, again ensuring a uniform 120-s duration for all trainees. The EEG recording was conducted using an Enobio 8 5G wireless device (Neuroelectrics, Cambridge, MA, USA). Eight channels were used—Cz, Fz, P7, P8, P3, P4, O1, and O2—following the international 10–20 Montage system for electrode placement [18,19]. To facilitate this, the trainees were asked to wear a head cap embedded with electrodes before they commenced the tasks. Data were recorded at a sampling rate of 500 Hz. Any electrical line noise (50 Hz) was filtered out. The time-points for the EEG recording mirrored those of the EMG recording, thus maintaining consistency in our data collection approach. Before analysis, EEG data were filtered using a bandpass filter from 0.1 to 44 Hz in Matlab (24.2; The Mathworks, Natick, MA, USA). Subsequently, the maximum power of the alpha frequency band was extracted from the power spectra. Data were analyzed using a mixed-model (2 (group: O&G, GS) × 4 (simulated tasks: (LCN, HEC, BMC, KNOT)) ANOVA, with a subsequent Šidák post hoc test to locate any differences. In this context, group (O&G vs. GS) was the between-subject factor and task (LCN, HEC, BMC, and KNOT) was the within-subject factor. No covariates were included. Cohen’s *d* quantified the between-group effect size using the mean difference divided by the pooled standard deviation. Data analysis and figure preparation were conducted using GraphPad Prism 10 (GraphPad Software 2365 Northside Dr. Suite 560 San Diego, CA 92108, USA). All data were expressed as mean ± standard deviation (SD) unless otherwise stated. Significance was set at *p* < 0.05.

Outcomeij = β0 + β1 × GroupGS + β2 × TaskHEC + β3 × TaskBMC + β4 × TaskKNOT + β5 (GroupGS × TaskHEC) + ⋯ + (1∣Subjecti) + εij

## 3. Results

**Figure 1:**
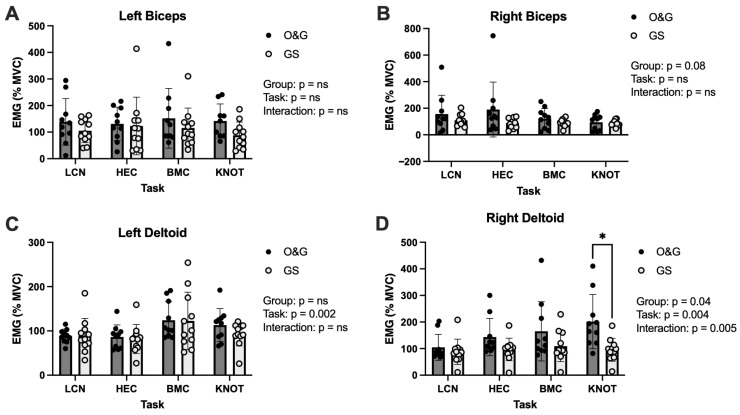
EMG (% maximal voluntary contraction) activity of the biceps and deltoids in O&G and GS trainees when completing core simulated laparoscopic tasks. (**A**): left biceps, (**B**): right biceps, (**C**): left deltoid, and (**D**): right deltoid muscles. LCN: laparoscopic camera navigation; HEC: hand–eye coordination; BMC: bimanual coordination; and KNOT: laparoscopic suturing and intracorporeal knot placement. Data are displayed as mean ± SD. * *p* < 0.05.

Between-group differences for our primary outcome variable, EMG, from the left or right biceps or the left deltoid did not reach significance at *p* < 0.05 (Figure 1A–C). However, between-group effect sizes for the left biceps were *d* = 0.46, 0.69, 0.53, and 0.21 for the LCN, HEC, BMC, and KNOT tasks, respectively. Between-group effect sizes for the right biceps were *d* = 0.50, 0.10, 0.40, and 0.90 for the LCN, HEC, BMC, and KNOT tasks, respectively. Between-group effect sizes for the left deltoid were *d* = 0.02, 0.16, 0.03, and 0.65 for the LCN, HEC, BMC, and KNOT tasks, respectively.

Left deltoid EMG elicited a main effect of task (*p* = 0.002) (Figure 1C) and post hoc analysis revealed significantly greater EMG activity in BMC vs. HEC but only in the O&G group (*p* = 0.016). There was an effect of group (*p* = 0.04) in the right deltoid where EMG activity was higher in O&G than GS. Furthermore, there was a group x task interaction (*p* = 0.005) and a significant effect of task (*p* = 0.004) for right deltoid EMG (Figure 1D). Between-group differences for the right deltoid were *d* = 0.37, 0.79, 0.64, and 1.18 for the LCN, HEC, BMC, and KNOT tasks, respectively.

**Figure 2:**
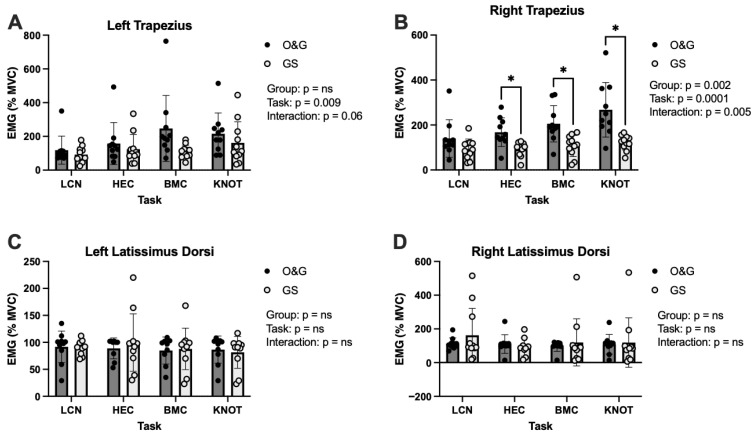
EMG activity of the trapezius and latissimus in surgical specialties when completing different laparoscopic tasks. (**A**): left trapezius, (**B**): right trapezius, (**C**): left latissimus dorsi, and (**D**): right latissimus dorsi muscles. LCN: laparoscopic camera navigation; HEC: hand–eye coordination; BMC: bimanual coordination; and KNOT: laparoscopic suturing and intracorporeal knot placement. Data are displayed as mean ± SD. * *p* < 0.05.

Between-group differences for our primary outcome variable, EMG, from the left trapezius (*p* = 0.137), left latissimus dorsi (*p* = 0.817), and the right latissimus dorsi (*p* = 0.559) did not reach *p* < 0.05 (Figure 2A,C,D). Between-group effect sizes for the left trapezius were *d* = 0.40, 0.33, 0.93, and 0.45 for the LCN, HEC, BMC, and KNOT tasks, respectively. Between-group effect sizes for the left latissimus dorsi were *d* = 0.06, 0.27, 0.01, and 0.19 for the LCN, HEC, BMC, and KNOT tasks, respectively. Between-group effect sizes for the right latissimus dorsi were *d* = 0.43, 0.33, 0.22, and 0.01 for the LCN, HEC, BMC, and KNOT tasks, respectively.

Left trapezius EMG was significantly different dependent upon task (*p* = 0.009) (Figure 2A) and post hoc analysis revealed significantly higher EMG activity in BMC vs. HEC but only in the O&G group (*p* = 0.049). There was an effect of group (*p* = 0.002), task (*p* = 0.0001), and group x task interaction (*p* = 0.005) for the right trapezius muscle EMG (Figure 2B). Post hoc analysis revealed differences between O&G and GS trainees in HEC, BMC, and KNOT. Between-group effect sizes for the right trapezius were *d* = 0.70, 1.25, 1.25, and 1.32 for the LCN, HEC, BMC, and KNOT tasks, respectively.

There were no between group differences for our secondary outcome variable, alpha power determined using EEG, between groups for the LCN task, the HEC task, the BMC task, or the KNOT task (Table 1), at the *p* < 0.05 level (Table 2). However, there were small (*d* = 0.48) to large (*d* = 0.84) differences between groups, suggestive of greater cognitive demand in O&G trainees, but the differences were not significant and should be interpreted cautiously.

## 4. Discussion

Our findings suggest that O&G trainees experience greater muscle activation during the same simulated laparoscopic tasks as GS trainees. Higher muscle demands could lead to motor control reductions in the short term and musculoskeletal disorders in the long term, impacting sick leave and career longevity [20,21]. Indeed, O&G trainees may therefore be at a higher risk of musculoskeletal disorder during their career compared to GS trainees due to the adverse consequences of increased muscular activity during laparoscopic abdominal procedures. It is thus important to consider and address the root cause of the observed disparity. One possible explanation is the decreased exposure to laparoscopic surgery that O&G trainees have compared to GS trainees; they may adopt less optimal ergonomics, which impacts muscle activation. In terms of training pathways in the UK, GS training in the UK lasts eight years (CT1–ST8), with laparoscopic exposure starting early. Conversely, O&G training is seven years (ST1–ST7), but ST1–ST5 are mainly obstetrics-focused, and only those pursuing a gynecological pathway receive intensive laparoscopic training in the final two years. Ultimately, this results in an earlier and greater laparoscopic case volume for GS trainees compared to O&G [22], and subsequently better performance in simulated laparoscopic tasks in GS trainees compared to O&G [12]. 

Alternatively, the GS group had a trend of more males (8 vs. 4, *p* = 0.068), who tend to be taller and therefore more likely to employ different strategies to reach. While sex-based ergonomic differences have been suggested in the literature, our study was not powered to confirm this, so this explanation remains speculative, but differences in surgeon height could lead to postural compensation. This could be important as research in the field has already identified that maintaining ergonomically sub-optimal static positions is a major cause of musculoskeletal strain [23]. Considering the lack of demographic difference between the two trainee groups, a suggestion for the difference in muscle activation could be differences in ergonomics [24]. Indeed, our earlier study demonstrated that O&G trainees had inferior performance compared to GS trainees on the same simulated laparoscopic tasks [12]. The findings of the current study indicate that this disparity may be partially attributable to differences in muscle recruitment and ergonomics. One solution may include increasing the height of the operating table or improving the ergonomic design of surgical tools [25]. The simulated nature of these tasks means that the real-world generalizability of the findings are limited, as real-world surgery involves the patient outcomes being the number one priority. The surgeons’ ability to adhere to this creates a dynamic set of variables from surgery to surgery depending on the needs of the patient. It may also be important to note that although we observed greater musculoskeletal demand within O&G trainees compared to GS trainees, we do not know how this translates to actual musculoskeletal injury in terms of long-term outcomes. Research within this field would benefit from cross-sectional studies that also take a longitudinal approach to follow up on surgeon musculoskeletal health to create a more accurate picture of injury risk. Due to the cross-sectional nature of this study, there is a risk of bias within this procedure, as there is no blinding of the trainees, potentially leading them to alter their technique. Our findings are also limited by the inherent methodological constraints of EMG and EEG. Indeed, they are susceptible to movement artefacts and noise that impact the data. Combined with the simulated task limitations, these affect the overall ecological validity of the findings.

In terms of the cognitive aspect, our analysis of alpha activity did not show any differences at the *p* < 0.05 level between the two groups across tasks. However, given EEG was our secondary outcome variable, this study was not powered to detect differences in EEG. Therefore, we interpreted these findings using a combination of *p* values and effect sizes. Between-group deviations were observed, which ranged from small (KNOT task) to large (HEC task), suggesting greater cognitive demand in O&G trainees. However, further research with a larger sample size is needed to validate these preliminary findings. An alternative explanation is that cognitive loads were consistent across surgical specialties during these tasks. Moreover, alpha activity, while commonly associated with cognitive attentional demands, might not be the maximally sensitive measure in the setting of these motor-skill-heavy tasks. Other EEG frequencies, such as beta (associated with active concentration and motor control) or gamma waves (linked to cognitive processing and integration of sensory information), could potentially be more indicative of cognitive differences in this context. Beta activity in particular is associated with motor planning and execution and is linked to motor learning. Therefore, in the trainee context, beta activity might present a more sensitive index of the neural basis of visuomotor learning and surgical skill deployment, and would provide a different insight relative to the comparison of cognitive skills indexed by alpha activity. Future work should explore in greater detail the neural basis of surgical skill development and the different neural signatures that may underlie different elements of surgical practice. It would also be informative for future work to link the brain to behaviour, and explore how, e.g., beta activity correlates with NASA TLX scores to understand how subjective measures of effort relate to objective neural data. In summary, further research is needed to validate our findings; identify risk factors for musculoskeletal injury, such as the impact of size and gender; and evaluate interventions to promote musculoskeletal health and prevent occupational injury. Moreover, future work should evaluate the effectiveness and cost-effectiveness of ergonomic training programmes, particularly for O&G trainees, in reducing physical demand. 

## 5. Conclusions

Collectively, our findings suggest that O&G trainees experience greater musculoskeletal demands than GS trainees when performing several core laparoscopic skills. These differences are reflected in the greater EMG activity of the right trapezius and right deltoid muscles and therefore suggest that specialty-specific training may influence ergonomics during laparoscopic tasks. The findings highlight the importance of incorporating ergonomic training into training curricula to mitigate long-term musculoskeletal disorders and promote surgeon wellbeing.

## Figures and Tables

**Table 1:** Characteristics of O&G and GS trainees. Data are presented as number (%) or mean ± SD.

Trainee Characteristics	O&G (*n* = 10)	GS (*n* = 10)	*p* Value
Female	6 (60)	2 (20)	0.068
Male	4 (40)	8 (80)	
Mean age (years)	32 ± 3	33 ± 4	0.77
Body mass (kg)	73 ± 14	83 ± 9	0.22
Height (metres)	1.69 ± 0.06	1.75 ± 0.06	0.12
BMI (kg/m^2^)	26.1 ± 0.97	26.8 ± 0.58	0.73
Junior grade	5 (50)	6 (60)	0.65
Senior grade	5 (50)	4 (40)	
Activity level			
Active	6 (60)	6 (60)	1.0
Moderate	3 (30)	3 (30)	
Inactive	1 (10)	1 (10)	
Presence of musculoskeletal pain over previous 12 months	6 (60)	8 (80)	0.33

For O&G, ST5 stages of training were classed as junior. Senior GS trainees were categorized as those with a specialty focus, excluding breast surgery. Senior O&G trainees were categorized as trainees within the final 2 years of their training, as described previously. Activity level was self- reported by surgeons during the activity questionnaire. Presence of musculoskeletal pain was measured using the Nordic Musculoskeletal Questionnaire.

**Table 2:** Comparison of alpha power between O&G and GS trainees during the different simulated tasks.

Task	O&G	GS	Group Comparison *p* Value, *d* Value
Laparoscopic camera navigation	16.2	20.7	0.421, 0.53
Hand–eye coordination task	13.5	15.6	0.714, 0.84
Bimanual coordination task	11.3	14.1	0.639, 0.59
Intracorporeal knot placement task	10.9	12.9	0.740, 0.48

## Data Availability

This study was prospectively registered at ClinicalTrials.gov (NCT05116332), and data will be made open.
